# Measuring trust in healthcare with instruments developed in different disciplines – A scoping review

**DOI:** 10.1177/09697330241272806

**Published:** 2024-08-10

**Authors:** Venla Karikumpu, Arja Häggman-Laitila, Anja Terkamo-Moisio

**Affiliations:** 205537 University of Eastern Finland; 60655 University of Helsinki; 205537 University of Eastern Finland

**Keywords:** Healthcare, leadership, measurement, organization, peers, trust

## Abstract

**Background:**

Trust is a key character at organizational level. Understanding the level of trust with timely relevant instrument is a significant process to capture the level of trust beyond organizational changes in healthcare.

**Objectives:**

To gather, assess, and synthesize the items of instruments evaluating trust in healthcare organizations.

**Design:**

Scoping review methodology.

**Methods:**

The literature search with deductive-inductive content analysis. The data were charted from articles that involved the use of trust instruments in healthcare organizations.

**Data Sources:**

Search from eight databases was updated in January 2024 and included peer-reviewed articles published between 2010 and 2023.

**Results:**

A total of 13 instruments were found measuring trust in the organization, trust in the leader, and trust among peers in healthcare. The items of instruments about trust in the organization included strategic and operational cultures. The trust in the leader consisted of competence, consistency, openness, appreciative acceptance, and loyalty and risk, while instruments about trust among peers included dimensions of moral partnership, common interest, and competent peers.

**Conclusions:**

Comprehensively measuring trust in the leader, trust in the organization, and trust among peers is significant due to the multifaceted dimension of trust. Measuring trust offers a possibility to recognize the working relationships and cultures in healthcare organizations.

## Introduction

Nursing literature^1–3^ states that trust is one of the core values in nursing ethics. Based on its attributes, it has a normative value and a moral dimension. The attributes that are included in the definitions of trust are confident reliance on others’ competence and willingness to look after good will rather than harm, a willingness to engage in a relationship while accepting one’s vulnerability and the existence of a risk and uncertainty in the joint actions and decisions. It also includes the expectation that involved people will not seek to take advantage of others even if they have such opportunities.^1–3^

Although the concept of trust in healthcare organizations and nursing has been of interest for decades,^1,2,4,5^ the researchers have not agreed on a uniform definition of it^2^ nor a multidimensional instrument for the context of healthcare has been developed.^3^ The knowledge of the ways, how the concept of trust has been operationalized in the empirical research in healthcare and nursing is fragmented. This study investigated the instruments that have been used for measuring trust in the context of healthcare, presenting an overview of the operationalization of trust in healthcare organizations. The goal was to produce knowledge that could be utilized in healthcare to measure impersonal and interpersonal trust on employees’ and leaders’ perspectives.^6^ To our knowledge, there are no previous studies on the instruments of trust in this context.

## Background

Being a professional means a commitment to professional norms and use own competencies to meet the others’ needs. This can be successful only if the different stakeholders trust each other. Professionals have different levels of power based on their specialized knowledge and on a control over its use. Trust is the mean to overcome this power asymmetry in professional relationships.^2^ Trust occurs in human relationships and on different network levels. However, trust has been found to be fragile, once lost, it has been described almost impossible to rebuild. In nursing, trust has been described on interpersonal and impersonal levels.^1–3^

Interpersonal trust occurs in nursing within the context of nurse-patient relationship and among members of healthcare teams, the first mentioned being widely studied.^1,2^ For example, recent studies have focused on patients’ trust of the clinician and/or organization or clinicians’ trust of the patient^7–9^ with an aim at improving the quality of healthcare.^10^ The research tradition of the trust among members of healthcare teams is sparse, although over couple decades ago Peter and Morgan^1^ already highlighted its importance. Healthcare professionals are dependent on one another for their clinical decisions and conducting care, preserving patient safety and secure work environment.^1^ The healthcare sector’s hierarchical and paternalistic roots may challenge the experiences of trust in health care teams.^11^

Impersonal trust occurs within organizations.^2^ It is based on the web of the relationships formed by nurses, other healthcare professionals, and managers. Organizations earn impersonal trust by managing costs, providing secure and comfort work environment and establishing methods to identify the different stakeholders’ needs. Impersonal trust is a mechanism of power that helps to manage the complexity of the caring environment and to distribute resources in a justified manner. Power imbalances, financial constraints, organizational policies, and restrictions may challenge impersonal trust.^2^ Nurses face these challenges, for example, in the situations where they attempt to behave morally in a system in which they have little power.^1^ Impersonal trust extends beyond patients’ beds to broader levels of institutions and systems. It is affected by cultural, political, and historical contexts.^1,2^ Impersonal trust is also poorly empirically identified in the context of healthcare and nursing.

In a clinical context, trust in the leader enhances productivity at work by improving efficiency and enhancing quality of services and care.^12–14^ It also increases collaboration among stakeholders^15^ and effective performance of teams.^11^ Furthermore, trust in the organization increases employees’ satisfaction, commitment, and well-being at work.^16^ The impacts of trust on patients’ care quality and effectiveness and staff outcomes have also been identified in the health sector similarly as in other organizations.^4^ Trust is a dynamic, time-specific and context-sensitive concept,^10^ which should be measured within various levels.^17^ It is further to notice, that appearance, advancement, and maintenance of trust are not axioms.^11^ Due to the major impacts of trust at the organizational level, it should be evaluated systematically^11^ with an instrument that acknowledges its interpersonal and impersonal levels. Systematic evaluation provides knowledge for the development of the leadership and work communities in healthcare services.

### Objectives

The purpose of this study was to gather, assess, and synthesize the items of instruments evaluating trust in the healthcare organizations. The research questions were:

• What kind of items are included in the instruments evaluating trust in the organization?
• What kind of items are included in the instruments evaluating trust in the leader?
• What kind of items are included in the instruments evaluating trust among peers?

## Methods

### Design

We employed a scoping review methodology.^18^ This review is reported in accordance with the Preferred Reporting Items for Systematic Reviews and Meta-Analyses (PRISMA) Extension for Scoping Reviews,^19^ and with checklist (Supplemental file).

### Search methods

We conducted the first search in September 2022 and focused on psychometrically tested instruments of trust in the healthcare organization. Because this literature search yielded no results, we refined the search strategy with the support of an information specialist. We then conducted the second literature search in December 2022 via eight databases: Medic, PsycINFO, Medline (Ovid), SocINDEX, PubMed, CINAHL, Web of Science, and Scopus. The search terms included trust-related terms (see Table 1) combined by Boolean operators in both Finnish and English. We searched by adopting inclusion and exclusion criteria (see Table 1). Searches were limited to peer-reviewed articles published between March 2010 and December 2022 in Finnish, English, or German. This search was updated in January 2024 to catch year 2023, and one more article was included in the review.

**Table 1.** Search strategy with databases, search terms, and inclusion and exclusion criteria.

Databases	Search terms	Inclusion criteria	Exclusion criteria
Medic	luottamu* AND terveydenhuol*	Trust in the organization	Trust not defined or measured
PubMed	trust* AND ‘health care’ AND (leadership OR management) AND NOT (patient* OR client*)		Trust subscale with few items or other instrument used in analysis
Scopus		Context of healthcare	Items not available
Web of Science			Trust outside the leader or the organization
CINAHL		Empirical study designs	Other context than healthcare
PsycINFO			Reviews, meta-analyses, cases, or qualitative studies
SocINDEX		Includes instrument/tool/measure of trust	Language not in English, German, or Finnish

### Search outcome

**Figure 1.**
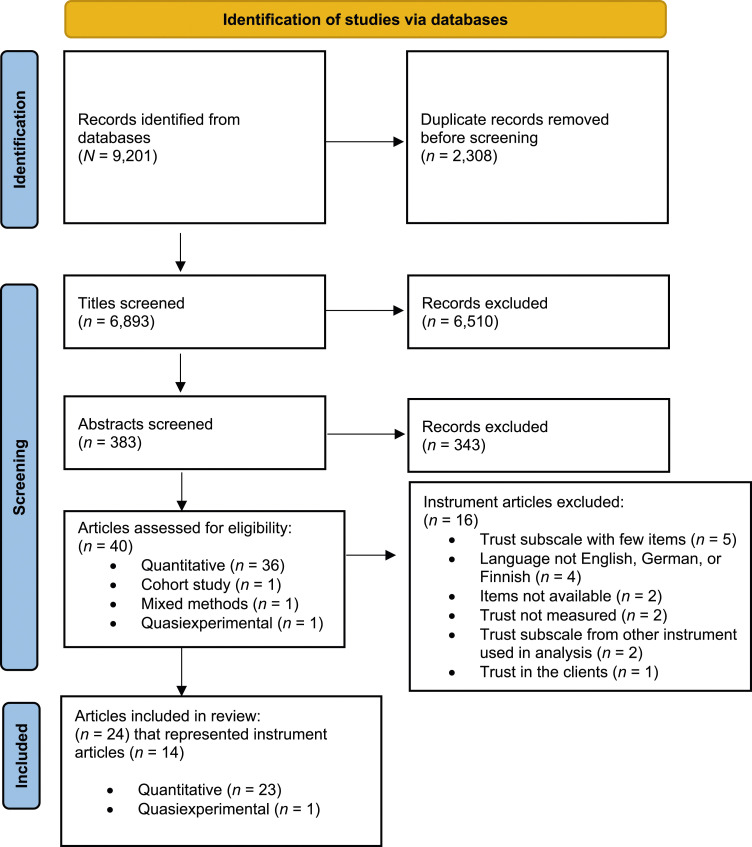
PRISMA Flow Chart of Search Results*. Note*. Adapted from Moher et al.^23^

The literature search resulted in 9201 titles overall, which we transferred into Covidence. After duplicates (*n* = 2308) were removed, titles (*n* = 6893), abstracts (*n* = 383), and full-text articles (*n* = 40) were independently screened and assessed by two authors (AA, BB). As Figure 1 shows, the results of the screening process were compared and discussed in each phase until a consensus was achieved.^19^

Thereafter, we searched the reference lists from the 40 full-text articles and then listed instruments (*n* = 30) utilized in those articles. Furthermore, we searched databases and university libraries to find utilized instruments. We excluded four instruments based on the language criteria and two instruments based on the missing original publication and items (see Figure 1). In addition, the original publications of two instruments were not retrievable, so we used secondary sources.^20,21^ Furthermore, one instrument article was retrieved directly from the author.^22^ The final data consisted of 24 articles presenting a total of 14 instrument articles, which evaluated trust in the context of healthcare.

#### Quality appraisal

Due to the diversity of the final data (scientific articles, book chapters, and secondary sources), quality appraisal did not take place, and we chose a scoping review as the method for this article.^24^ The authors (AA, BB, CC) discussed and evaluated the eligibility of the final data.

### Data charting and analysis

The data were charted in a matrix by one author (AA), while other author (CC) verified the data for accuracy.^19^ The data was structured by focus of trust: in the organization, in the leader, and among peers. The matrix included information about the study design, the base of instrument design, the study context, the secondary source, the structure of the instrument, the number of sum variables and items, and information about the reliability of the study and instrument when reported. Based on the research question, we analysed data with deductive-inductive content analysis^25^ by employing Atlas.ti software. After reading instrument articles, we identified and separated items of the instruments to the main categories (*n* = 3), based on the research questions. These main categories were trust in the organization, trust in the leader, and trust among peers. Original expressions (*n* = 207) in these main categories were first condensed resulting in 97 simplified expressions. These were then organized based on their similarities and differences in subcategories (*n* = 38), which were named based on their content. The subcategories were grouped into upper categories (*n* = 10) and named based on their content. (Table 2) This deductive-inductive content analysis was discussed in all stages within the research group until a consensus was achieved.^26^

**Table 2.** Example of the deductive-inductive content analysis.

Analysis stage	Example of analysis process
Item (original expression)	I trust that my fellow management team members express their true feelings about important issues.^27^
Simplified expression	Free expression of feelings and courage to discuss mistakes
Subcategory	Emotions in interaction
Upper category	Moral partnership
Main category	Trust among peers

## Results

### Description of the data

The data comprised 14 articles reporting a total of 13 instruments measuring trust entirely or partly in the leader (*n* = 9), in the organization (*n* = 4), and among peers (*n* = 4), as shown in Table 3. The oldest instrument was published in 1962^21^ and the newest in 2015^28^ while most were published in the 1990s (*n* = 7) or from 2003 to 2005 (*n* = 4). The studies were conducted in the United States (*n* = 9), Australia (*n* = 2), Canada (*n* = 1), Israel (*n* = 1), the UK (*n* = 1), Poland (*n* = 1), and across Europe (*n* = 1). The instruments collected answers by Likert scale, which included either five stages^20,21,29–32^ or seven stages.^22,27,28,33–36^ One instrument did not report the form of reply to the items.^37^

**Table 3.** Description of the instruments measuring trust with different dimensions in healthcare, theoretical background of trust, and method of instrument development.

Authors	Name of the instrument/Number of items	Development discipline	Theoretical background	Method of instrument development	Dimensions of trust			Reliability
					Organization	Leader	Peers	
Anand et al.^21^	Trust in Leadership/5	Not reported	Stodgill (1962)	Not reported		X*		Cronbach alpha 0.91 (incl. four items)
Cook and Wall^34^	Interpersonal Trust at Work/12	Industry	Not reported	Interview for employees		2	2	Coefficient alpha range 0.80–0.85
Podsakoff et al.^37^	Subscale: Trust/Loyalty in Leader/6	Industry	Two items from Cook and Wall (1980)	Not reported		X*		Coefficient alpha 0.90
Mishra and Mishra^27^	Mutual Trust/16	Industry	Not reported	Literature search and interview for managers			X*	N/A
Robinson^30^; Robinson and Rousseau^31^	Trust Scale/7	Business	Trust identified by Gabarro and Athos (1976)	Not reported	1			Cronbach alpha 0.93
McAllister^36^	Interpersonal Trust/11	Business	Earlier instruments of interpersonal trust (Cook & Wall, 1980; Johnson-George & Swap, 1982; Rempel et al., 1985; Rotter, 1971)	Analysis of expert evaluations			2	Cronbach alpha range 0.89–0.91
Cummings and Bromiley^33^	The Organizational Trust Inventory/62	Business	Trust as interpersonal (Helgeson, 1994; Wrightsman, 1991), intergroup (Zander, 1994), organizational (Bradach & Eccles, 1989; Gambetta, 1988; Granovetter, 1985; Hosmer, 1995), societal (Lewis & Weigert, 1985), or behavioural and belief-based (Shoda et al., 1994).	Groups of doctoral students	3			Composite reliability range 0.78–0.96
Akkaya^20^	Organizational Trust Inventory/12	Not reported	Nyhan and Marlowe (1997)	Not reported	1	1		Coefficient alpha range 0.95–0.96
Ferres and Travaglione^22^	Workplace Trust Survey/32	Healthcare	Development by researcher, concerning items from Cook and Wall (1980)	Focus group with critical-incident method, similar to Buss and Craik (1983) act-frequency analysis. Based on grounded theory.	1	1	1	Coefficient alpha range 0.89–0.96
Gillespie^35^	The Behavioural Trust Inventory/10	Not reported	Trust models by Mayer et al. (1995) and Zand (1972)	Not reported		2		Alpha range 0.91–0.92
Tzafrir and Dolan^32^	Trust Questionnaire/16	Business	Not reported	Interview for employees and managers with critical-incident method, similar to Buss and Craik (1983) act-frequency analysis		3		Coefficient alpha 0.92
Mayer and Gavin^29^	Trust Scale/10	Industry	Four items from Mayer and Davis (1999)	Six items developed with employee focus groups		2		Coefficient alpha range 0.72–0.81
Paliszkiewicz et al.^28^	Subscale: Trust Management/10	Various	Trust from psychology (Johnson-George & Swap, 1982; Rotter, 1971; Simpson, 2007), sociology (Lewis & Weigert, 1985; Yamagishi et al., 1998; Molm et al., 2000), and management (Mayer et al., 1995; McAllister, 1995; Dirks & Ferrin, 2002; Lewicki et al., 2006; Colquitt et al., 2007; Kramer & Lewicki, 2010; McEvily, 2011; Paliszkiewicz et al., 2014)	Not reported		X*		Cronbach alpha 0.94

*Note.* *X marks the missing amount information of the sum variables.

Eight of the instruments were multidimensional, and one was clearly one-dimensional, while the precise information of dimensionality was missing from the remaining instruments. The most frequent dimension was trust in the leader (*n* = 9), which was once separated into trust in plant manager and trust in top manager.^29^ The other two dimensions, trust in the organization and trust among peers, were included in four instruments (Table 3).

The original instruments were all employed in the context of healthcare, but developed in different disciplines (Table 3), which were business (*n* = 5) and industry (*n* = 4), whereas some were unknown (*n* = 4). One instrument was developed in the context of healthcare.^22^ Four articles reported instrument development and five testing of an instrument with cross-sectional designs. One instrument was presented in a book chapter,^33^ and two in longitudinal studies.^30,31^ Furthermore, access to items by Nyhan and Marlowe^20^ as well as Stodgill^21^ instruments was available only from secondary sources. The theoretical background of the instruments was not reported in five articles whereas earlier trust models and theories were used variously in three instrument articles (see Table 3). Some of the items for developed instruments (*n* = 5) were based on earlier trust instruments: Podsakoff et al.^37^ used two items, and McAllister^36^ used one item from Cook and Wall^34^ The duplicates of these items were excluded from the analysis. Ferres and Travaglione^22^ also used items (*n* = 10) from Cook and Wall,^34^ but these items were analysed in this study due to their altered spelling. Interviews were conducted in the development process of five instruments, of which two used critical-incident methods, and one used a literature search. Also, two instruments were developed with the method of expert evaluations. Furthermore, six articles did not report the method of instrument development (Table 3).

### Items in the instruments about trust in the organization

Trust in the organization was operationalized with 84 items, and we divided them into *strategic* and *operational cultures of organization*. Strategic culture of organization was operationalized in the instruments as an integrated organization consisting of reciprocal trust in all organizational levels.^20^ In that case, it was possible to evaluate credibility and sincerity in the organization.^33^ Also, strategic culture included an employee’s belief in the organization’s integrity. One example item of this was ‘I believe my employer has high integrity’.^31^ Furthermore, strategic culture was operationalized with all actors, individuals, or teams pursuing and behaving for the common interest of the organization instead of concentrating on the weaknesses of the organization or their own benefits. This was operationalized, for example, as ‘We think that the people in __ manipulate others to gain a personal advantage’.^33^ The strategic culture referred to the common vision of the organization with realistic goal-orientation. Also, it included abilities of the organization and commitment to agreed goals. Ultimately, it included a common belief of the success of the organization and its projects.^22,33,35^ An example item was ‘I have positive feelings about the future direction of X’.^22^

Operational culture of organization was operationalized as the organization’s open policy with a respectful attitude towards employees.^31,33^ Discussions in the organization were composed of reciprocity, sharing knowledge, honesty, approachability, and justice processes.^22,33^ One example of this was ‘I feel that information can be shared openly within X’.^22^ Furthermore, it included flexible timetables and loosening of control.^33^ More closely, respectfulness referred to a supportive environment, fair treatment, rewarding, and positive motives and intentions towards employees.^20,22,31^ These concepts were rationalized, for example, as ‘My level of trust that this organization will treat me fairly is_’.^20^ Operational culture comprised the operationalization of teams, which were engaged for partnership with other teams. In such cases, the members of an organization depended on each other and shared an atmosphere of commitment. This was operationalized, for example, by the item ‘We think X keeps the spirit of an agreement’.^33^

### Items in the instruments about trust in the leader

Trust in the leader was operationalized with 78 items, which we divided into *competence, consistency, openness, appreciative acceptance*, and *loyalty with risk*. Competence included the leader’s knowledge of work, abilities to complete the role, and skills including technical competence.^20,28,32,34,35^ For example, competence was operationalized as ‘Rely on your leader’s task-related skills and abilities’.^35^

Consistency included the leader’s consistent work performance, which was described as a commitment to the assignment, availability, and completing work efficiently and without complaints.^20,22,28,32,34,37^ Consistency was operationalized, for example, as ‘I believe that my manager follows words through with action’.^22^

Openness was operationalized as an interaction wherein the leader gives a possibility to the employees to discuss their personal issues and possible difficulties with the leader.^29,32,35^ This was operationalized as ‘Discuss how you honestly feel about your work, even negative feelings and frustration’.^35^ Furthermore, openness was the employees’ feelings that the leader’s interactions were consistent and reliable and that the leader was listening authentically.^22,28^ Also, it was described as a reciprocal, open interaction without fear of disadvantages.^29^ In that case, both parties were waiting for open information,^32^ especially the employees from the leader.^20,21^ One example item measuring openness was ‘If X asked me for something, I responded without thinking about whether it might be held against me’.^29^

Appreciative acceptance was described as employees’ feelings about the leader’s acceptance and empathy towards them while they were loyal to the leader.^28,32,34,37^ In that case, employees could be sure about the leader’s broad-mindedness about their work^20,34,35^ and challenges therein.^21^ Furthermore, it included reciprocal support especially in emergencies and taking care of others.^21,29,32,34,35,37^ Also, it was described as a respectful and fair behaviour from leaders while the leader was responding to the employees’ needs.^21,34^ One example item of this was ‘The Leadership Team responds well to my concerns’.^21^ Moreover, it included consistent actions from both parties, without offending the organization.^32^ This concept was reflected in a safe emotional atmosphere, wherein everyone felt safe to authentically express emotions without fear of others’ reactions.^29,32^ An example item to measure this was ‘I am afraid of what ___ might do to me at work’.^29^ Appreciation was also operationalized as a reciprocal self-esteem and respect of one’s wishes and perspectives as well as a feeling of the leader’s appreciation for employees’ work input.^22,29,32^ For example, one item stated ‘Employees/managers really look out for what is important to the managers/employees’.^32^

Loyalty with risk was stated as the employees’ feelings about the leader’s honesty and reliability with benevolence.^22,28^ Furthermore, it was the employees’ beliefs about the leader’s principles, which was operationalized as ‘A manager/leader’s honesty and principles contribute to elevated trust among people’.^28^ Moreover, both parties desired from the partnership a healthy attitude and fulfilment without the need for excessive supervision.^22,28,29,32^ This all was attached to an employee’s depending on the leader’s actions while both trusted each other and acknowledged reciprocal priorities.^28,29,35^ This was operationalized as ‘Depend on your leader to handle an important issue on your behalf’.^35^

### Items in the instruments about trust among peers

Trust among peers was operationalized with 44 items, which we divided into *moral partnership, common interest,* and *competent peers*. Moral partnership was operationalized as an ethical behaviour with honest intent to not monitor peers^22,27,34,36^ ad as the idea that peers had thorough and dedicated approaches to work.^22,34,36^ One example item of this was stated as ‘This person approaches his/her job with professionalism and dedication’.^36^ Moral partnership involved an appreciative attitude for another party’s work input and delight in, concern for, interest in, and respectful expectations for another party.^22,27,36^ Furthermore, peers were expected to have skills for open, reliable, and constructive social interactions especially when problems occurred at work.^22,36^ This was operationalized, for example, as ‘Given this person’s track record, I see no reason to doubt his/her competence and preparation for the job’.^36^ Furthermore, when those involved freely expressed emotions during interactions, they enabled each other to acknowledge their own mistakes.^27,36^ For example, one item measuring this stated ‘We have a sharing relationship. We can both freely share our ideas, feelings, and hopes’.^36^

Common interest consisted of supporting, helping, and sharing knowledge with peers.^22,27,34^ Encouraging common interests was important to the success of the organization in the future, where employees’ self-interests were subjugated to those of the organization. Common interest was operationalized, for example, as ‘I trust that my fellow management team members place our organization’s interests above their own’.^27^

Competent peers were operationalized to have professional and substantial working skills combined with keeping promises and acting consequently.^22,27,34,36^ One item measured this as ‘I trust that my fellow management team members take actions that are consistent with their words’.^27^

## Discussion

This scoping review synthesized the items of instruments evaluating trust in the organization, trust in the leader, and trust among peers in healthcare contexts. To our knowledge, this scoping review is the first one on the subject and thus deepens and clarifies the operationalization and appearance of trust in different organizational levels from the perspectives of employees. Our review produced a clear, multidimensional overview of the concept of trust, that has been acknowledged as a central value in nursing ethics.^1,2^ It is, however, to notice, that even when instruments enable periodical evaluation of trust, it’s subjective and dynamic nature require constant evaluation and consolidation, that should be taken into account in different levels of organization.^10^ The results of the current study provide concepts that may be utilized in the trust-related discussions and situations as well as in challenges concerning interpersonal relationships.

Trust in the organization consisted of strategic and operational cultures of the organization whereas items measuring trust in the leader and trust among peers were described with more human-related characteristics emphasizing reciprocal partnerships. This highlights the ethical perspectives such as benevolence, vulnerability and openness described in the previous literature.^1,2^ In some of the instruments, the complexity of trust was acknowledged^17,38^ but poorly named and operationalized. This was especially evident concerning measurements of trust among peers. For example, the content of the items in the Cummings and Bromiley^33^ instrument concerned trust among peers, but the authors defined the items by describing trust on an organizational or team level. Our examination focused on the perspective of employees and leaders. In order to comprehensively understand the complexity of impersonal and interpersonal trust in the organization, we need besides the employee perspective, also knowledge on the perspectives of caregivers and patients, keeping in mind the imbalanced power relationships.^1,2^

Trust as a reflexive concept changes over time while organizations do as well, so the topicality of the instruments and its items needs assessment. The utilization of earlier instruments resulting in this review as such is challenged due to their unilaterality, age, and various cultural contexts of the data.^6,17^ Timely, appropriate instruments of organizational trust are formulated in contexts other than healthcare^39^ or with a focus on one dimension of trust in the healthcare team.^38^ Therefore, we suggest that the results of this review be utilized in the instrument-development process in a healthcare context. Multidimensional instruments measuring trust are needed in healthcare,^38^ wherein leaders and employees are facing multiple challenges in a complex context.^10,11^ Measuring trust with validated instruments produces evidence-based knowledge of the state of trust in the organizational culture^40^ and for the development of it and organizational practices and change management. Regular measurement addresses the current trust level and indicates the best possible concrete interventions to strengthen it.^6^ Additionally, awareness of the current level of reciprocal trust increases the knowledge and building of trust, which have been acknowledged to be time-consuming.^9^ Furthermore, the increased trend of hybrid leadership highlights the importance of a trust-creating culture in healthcare^41^ and underlines the need for a robust instrument development.

The development of multidimensional trust instruments for employees in healthcare requires corroboration in close collaboration with healthcare organizations. The operational descriptions need to be phrased with healthcare specialists. Furthermore, to include psychometric properties for the validation, the instruments demand testing with healthcare leaders and employees.^6^ Thus, thoroughly developed instruments provide answers to the research questions in expected ways and provide valid responses,^42^ while the validation of instrument-development processes increases the feasibility of the instrument.^17^ To create or refine a current, context-derived instrument, there should be applied, qualitative research concerning trust in the nursing field.^17^

## Strengths and limitations

Several strengths of this study are that instruments in the data were utilized in healthcare sectors in different countries, we searched within eight databases, and the publication time frame was extensive. Another strength of this review is that we utilized a scoping review. It enabled us to observe instruments in a broad way when some of the data were not presented in peer-reviewed, scientific form^18^ and helped to avoid publication bias.^43^ The data were rich and enabled us to categorize trust into three dimensions. However, our review also has several limitations. For one, instruments in the data did not include a comprehensive report of the theory behind them, and the psychometric properties were introduced fragmentally, which challenged the reliability assessment of the instruments. Second, the instruments were outdated and developed mainly outside of healthcare in several other disciplines.

## Conclusion

This study provides an overview of the ways impersonal and interpersonal trust have been evaluated and operationalised in the context of healthcare. The results may be employed in future research and the trust enhancing development work within the organizations. Contents that hereby should be acknowledged are strategic and operational cultures along with human-related characteristics of trust that emphasize reciprocal partnership, benevolence, openness, and competence. Further research should specifically be targeted to the development of an instrument aimed to the context of healthcare.

## Supplemental Material

Supplemental Material - Measuring trust in healthcare with instruments developed in different disciplines - A scoping review Supplemental Material for Measuring trust in healthcare with instruments developed in different disciplines - A scoping review by Venla Karikumpu, Arja Häggman-Laitila and Anja Terkamo-Moisio in Nursing Ethics.
